# Facilitators of and Barriers to the Use of a Digital Self-Management Service for Diagnostic Testing: Focus Group Study With Potential Users

**DOI:** 10.2196/45115

**Published:** 2024-05-10

**Authors:** Kyma Schnoor, Esther P W A Talboom-Kamp, Muamer Hajtić, Niels H Chavannes, Anke Versluis

**Affiliations:** 1 Department of Public Health and Primary Care Leiden University Medical Center Leiden Netherlands; 2 National eHealth Living Lab Leiden University Medical Center Leiden Netherlands; 3 Zuyderland Sittard-Geleen Netherlands; 4 Department of Medical informatics University of Amsterdam Amsterdam Netherlands

**Keywords:** eHealth, usability, self-management, diagnostic test service, diagnostic, testing, test service, perspective, focus group, user need, user testing, implementation, qualitative, test result, laboratory test, laboratory result

## Abstract

**Background:**

Health care lags in digital transformation, despite the potential of technology to improve the well-being of individuals. The COVID-19 pandemic has accelerated the uptake of technology in health care and increased individuals’ willingness to perform self-management using technology. A web-based service, Directlab Online, provides consumers with direct digital access to diagnostic test packages, which can digitally support the self-management of health.

**Objective:**

This study aims to identify the facilitators, barriers, and needs of Directlab Online, a self-management service for web-based access to diagnostic testing.

**Methods:**

A qualitative method was used from a potential user’s perspective. The needs and future needs for, facilitators of, and barriers to the use of Directlab Online were evaluated. Semistructured focus group meetings were conducted in 2022. Two focus groups were focused on sexually transmitted infection test packages and 2 were focused on prevention test packages. Data analysis was performed according to the principles of the Framework Method. The Consolidated Framework for Implementation Research was used to categorize the facilitators and barriers.

**Results:**

In total, 19 participants, with a mean age of 34.32 (SD 14.70) years, participated in the focus groups. Important barriers were a lack of privacy information, too much and difficult information, and a commercial appearance. Important facilitators were the right amount of information, the right kind of tests, and the involvement of a health care professional. The need for a service such as Directlab Online was to ensure its availability for users’ health and to maintain their health.

**Conclusions:**

According to the participants, facilitators and barriers were comprehension of the information, the goal of the website, and the overall appearance of the service. Although the service was developed in cocreation with health care professionals and users, the needs did not align. The users preferred understandable and adequate, but not excessive, information. In addition, they preferred other types of tests to be available on the service. For future research, it would be beneficial to focus on cocreation between the involved medical professionals and users to develop, improve, and implement a service such as Directlab Online.

## Introduction

### Background

Society is changing, and the world is becoming increasingly digital [1]. Health care lags in digital transformation, despite the potential of technology to improve the well-being of individuals [1,2]. The COVID-19 pandemic accelerated the development and use of technology in health care, also referred to as eHealth, with more digital consultations and increased use of home monitoring [3,4]. Furthermore, the COVID-19 pandemic, among others, has increased the need and willingness of individuals to perform self-management [5-7]. In patients with chronic diseases, self-management strategies are often used to support patients in dealing with treatment and lifestyle changes [8]. In addition, self-management strategies can be used to support individuals with home diagnostic tests [9]. The concept of self-management aligns with this positive health definition: “health as the ability to adapt and self-manage in the face of social, physical, and emotional challenges” [10,11].

eHealth can be used in 3 stages of laboratory diagnostic testing. The first stage is triage and advice on diagnostic testing, the second stage is the testing itself (ie, at home or a facility), and the third stage is the communication of the test results to the user. A systematic review by Versluis et al [9] showed that web-based diagnostic testing services were positively evaluated and preferred over clinic-based testing. However, most of the evaluated services only offered tests to detect sexually transmitted infections (STIs) [9].

eHealth services can support self-management, for example, with web-based services that support behavior and lifestyle changes (eg, Liva Healthcare) [12] and with websites where individuals can obtain health information (eg, Thuisarts.nl) [13]. In addition, there are multiple apps to support patients with chronic conditions such as hypertension, diabetes, or lower back pain [14-16].

In the Netherlands, a web-based service called Directlab Online (Saltro, part of Unilabs) offers individuals direct access to laboratory diagnostic tests independent of a health care provider [17]. It is a so-called direct-to-consumer platform. Directlab Online gives individuals direct digital access to diagnostic testing based on a triage that aligns with medical guidelines. Unlike the services identified in the systematic review by Versluis et al [9], Directlab Online offers a variety of diagnostic tests, for example, diagnostic tests for STIs, COVID-19, and vitamin deficiencies, as well as testing for health-related questions concerning fatigue and the prevention of heart disease. The results and the information on the website can give individuals insight into their health, which could support and motivate them to adopt healthier behaviors [12]. In addition, it supports users to be better informed about their health without the interference of a health care professional, which can lead to more efficient and accessible care [18]. Packages to test the health of individuals align with the patient-centered care approach, which can lead to a better quality of care [19]. Patient-centered care aims to empower patients to take charge of their health and actively participate in their health care [20]. Another term used is person-centered care, which is similar but does not solely focus on disease-related aspects, aligning better with the positive health definition [21].

To completely harness the potential and significance of Directlab Online, prioritizing high-quality and user-friendly service is paramount. Delving into the barriers and facilitators individuals encounter when using the service could provide invaluable insights, facilitating the enhancement of its user-friendliness and effectiveness. For example, known factors in dermatology that could influence the uptake of a digital service are, among others, financial aspects and accessibility for a digital service [22]. In a study by Vergouw et al [23], facilitators of and barriers to digital services for older adults in primary care were researched. Nonfamiliarities with web-based environments appeared to be a barrier, and efficiency was seen as an important facilitator for using a digital service in primary care [23]. In the review of STI testing by Versluis et al [9], concerns regarding complicated language and data handling insecurities were also discovered for ordering an STI test on the web. To our knowledge, no research has been conducted on facilitators, barriers, and needs of a direct-to-consumer platform that offers direct access to multiple diagnostic tests and web-based results. Identifying the needs, facilitators, and barriers will help determine what is necessary to optimize the use and improve the implementation of those services. This can give insight into the potential future directions for developing such services.

### Objectives

This study aims to identify the facilitators of and barriers to using a service such as Directlab Online and to identify the needs regarding direct digital access to diagnostic testing. To achieve this, focus groups were conducted. Half of the focus groups focused on STI test packages and the other half on prevention test packages. STI tests and prevention test packages are the most ordered test packages on Directlab Online. The focus is on potential users, that is, those who have not used Directlab Online before, because we are interested in capturing people’s first impression of the service.

## Methods

### The Service: Directlab Online

**Figure 1:**
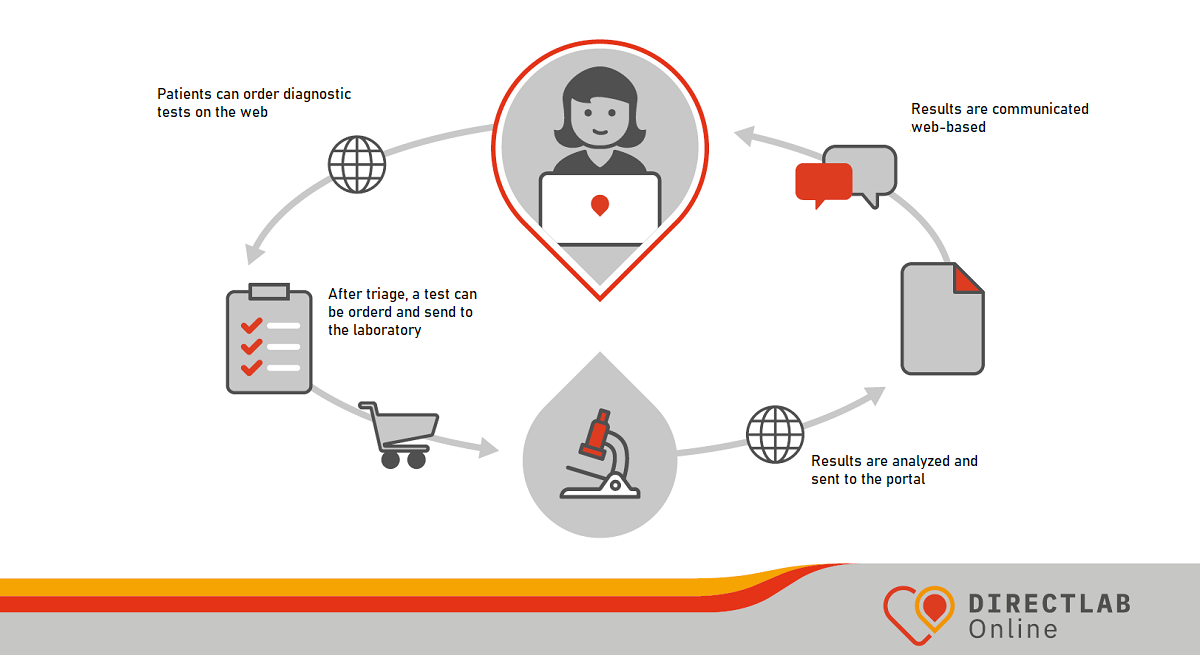
Stages of using Directlab Online.

Directlab Online is a Dutch, web-based service available for everyone, through which diagnostic tests can be ordered on the web [17]. The service was developed by a multidisciplinary innovation team of a diagnostic company (Saltro, part of Unilabs) and was launched in 2016 [24,25]. The process of using the service is presented in Figure 1. First, individuals undergo a web-based triage, based on medical guidelines, to determine whether the diagnostic tests are relevant and, if applicable, which tests are relevant. Second, individuals can order and buy associated tests. Depending on the diagnostic tests ordered, a self-sampling kit is sent to the individual’s home address, or an appointment is scheduled at a blood collection center or a laboratory for collecting a blood sample. Once the laboratory receives the collected specimen, high-quality analyses are conducted. The results of the tests are communicated through a web-based, secure patient portal. Furthermore, deviating results are communicated to the patient’s general practitioner but only if the patient has authorized it. The triage was based on medical guidelines, and the diagnostic test packages were developed in cocreation with general practitioners and tested by them and laboratory specialists referred to as health care professionals. Diagnostic test packages consist of different parameters for diagnostic testing. For example, a test package for cholesterol measures the following parameters: low-density lipoproteins, high-density lipoproteins, triglycerides, and total cholesterol. Multimedia Appendix 1 provides a complete overview of the test packages that could be ordered on Directlab Online during the focus group meetings. Table 1 provides an overview of the prevention and STI test packages that were part of the discussions with the focus groups.

**Table 1:** Test packages that are available on Directlab Online.

Category			Parameters
**Prevention tests**			
	Health checkup	Check total cholesterol^a^, low-density lipoproteins (LDL)^a^, high-density lipoproteins (HDL)^a^, triglycerides^a^, HbA_1c_^a^, and albumin and creatinine ratio^b^	
	Health checkup at home^c^	Measure parameters via self-sampling of blood: total cholesterol^d^, LDL^d^, HDL^d^, triglycerides^d^, HbA_1c_^d^, and albumin/creatinine ratio^b^	
	Cholesterol	Check total cholesterol^a^, LDL^a^, HDL^a^, and triglycerides^a^	
	Cholesterol at home^c^	Measure parameters via self-sampling of blood: total cholesterol^d^, LDL^d^, HDL^d^, and triglycerides^d^	
	Anemia	Check hemoglobin^a^, mean corpuscular volume^a^, ferritin^a^, and C-reactive protein^a^	
	Diabetes	Check glucose^a^ and HbA_1c_^a^	
	Healthy bones^c^	Check calcium^a^ and vitamin D^a^	
	Healthy kidneys^c^	Check creatinine^a^, glomerular filtration rate^a^, and albumin/creatinine ratio^b^	
	Thyroid check	Check thyroid function via thyroid-stimulating hormone^a^ and free T4^a^	
**Sexually transmitted infection tests**			
	Chlamydia	Check for chlamydia^e^	
	Gonorrhea	Check for gonorrhea^e^	
	HIV	Check for HIV^a^	
	Syphilis	Check for syphilis^a^	
	Hepatitis B	Check for hepatitis B^a^	

^a^Blood sample needed for diagnostics; HbA_1c_: hemoglobin A_1c_.

^b^Urine sample needed for diagnostics.

^c^These tests are not available any more on Directlab Online after the service update.

^d^Blood sample needed for diagnostics collected by self-sampling.

^e^Oral, anal, vaginal, or urine sample needed for diagnostic tests.

### Study Design and Participants

Focus group meetings were conducted with potential users of the service. As the Directlab Online service offers a wide variety of test packages, we focused on 2 specific categories (ie, prevention test and STI test packages). These test packages were ordered most frequently. Half of the focus groups focused on STI test packages, and the other half focused on the prevention test packages. The general inclusion criteria for the focus groups were speaking Dutch and not having used Directlab Online earlier. In addition, there were specific inclusion criteria to ensure that the sociodemographic characteristics of the participants in the focus groups were consistent with the characteristics of the target population of the test packages. Notably, a specific inclusion criterion for the focus group about STI testing was that the participants were aged between 18 and 30 years. The specific inclusion criterion for the focus groups about prevention test packages was that the participants were aged between 18 and 65 years. It is important to note that there were no specific health or disease requirements to participate. Focus group meetings were held until data saturation was reached.

### Ethical Considerations

The study was declared to not fall within the scope of the Dutch Medical Research Involving Human Subjects Act by the Leiden University Medical Center Medical Ethics Committee (N21.101).

### Procedure and Data Collection

The recruitment period started on October 25, 2021, and lasted until February 20, 2022. Participants were recruited via different web-based channels (eg, LinkedIn [LinkedIn Corporation] and Facebook [Meta platforms, Inc]). Individuals were invited to contact KS via email when interested. Then, KS sent them more information. In addition, questions were asked regarding their birth year and if they could understand Dutch. A few date options for web-based meetings were sent if the individual met the inclusion criteria. When individuals could participate, they received an email with the date and time, a link to the Zoom (Zoom Video Communications) platform where the meeting would be conducted (on the web), and a link to a web-based informed consent form, which they were asked to sign before participation. All participants had the right to withdraw at any moment. The focus group meetings occurred between January 10 and March 2, 2022, in the presence of MH and KS [26]. KS led the focus groups, and MH managed the time and assisted with technical issues. The focus group meetings were in a semistructured format, following a predefined topic list with open-ended questions to leave space for discussion (Multimedia Appendix 2). First, general questions were asked regarding using eHealth to see how familiar participants were with eHealth. Second, participants were provided 10 minutes to view the website of Directlab Online and navigate through the website on a computer or mobile phone; no further instructions were given. When time was up, questions were asked regarding the website in general (eg, the first impression, whether they needed help when using the website, and whether they found the website attractive). While navigating the website, they had the option to write down notes or vocalize their impressions, expressing their observations, preferences, and feelings about the website [27]. Third, participants were instructed to go through Directlab Online, do some triages, and look at their test advice. Notably, we allowed participants to navigate through the process as normal users would. Hence, they were required to peruse informational materials, undergo a triage process involving medical inquiries concerning their symptoms, and obtain guidance regarding testing. Subsequently, questions were asked regarding the triage service, facilitators of and barriers to using Directlab Online, and the participants’ needs for such a service. At the end of the focus groups, they received a digital gift card of €25 (US $27).

### Data Analysis

All focus group meetings were audio recorded for subsequent analyses and were transcribed (intelligent) verbatim. When the transcripts were completed, the audio records were deleted. Two reviewers, MH and KS, conducted the qualitative data analysis according to the principles of the Framework Method [28]. The Framework Method is a systematic and flexible approach commonly used for the thematic analysis of health research semistructured interview data [29]. The method combines deductive and inductive techniques, which align with the aim of the study to identify specific issues regarding the use of Directlab Online and leave space to identify needs and opportunities that have not been formulated a priori. First, open coding was performed independently by the 2 reviewers, KS and MH. The interview data were coded using the software Atlas.ti 22 (Atlas.ti 22 Scientific Software Development). Second, the codes were compared between the 2 reviewers. Third, the codes were grouped into categories, resulting in the analytical framework. Fourth, for identifying the facilitators and barriers, the Consolidated Framework for Implementation Research (CFIR) was used [30]. The framework is widely used for the content analysis of qualitative data regarding the factors influencing implementation success [30]. Furthermore, the framework is comprehensive and makes it convenient to systematically study a wide array of facilitators and barriers [31]. In addition, using this framework made it possible to compare the findings and transfer them to other implementation studies [32]. The CFIR is a theory-driven model and comprises five domains: (1) the innovation domain, (2) the outer setting domain, (3) the inner setting domain, (4) the individuals’ domain, and (5) the implementation process [30,33]. Identified facilitators and barriers were placed within the CFIR domains. Final themes were achieved via discussion and consensus between researchers KS and MH.

## Results

### Participant Characteristics

Data saturation was reached after forming 4 focus groups with 19 participants. The characteristics of the participants are shown in Table 2. The participants were aged 20 to 61 (mean 34.32, SD 14.70) years. The number of male (9/19, 47%) and female (10/19, 53%) participants was almost equal. The focus group meetings lasted around 90 minutes per group.

Age differed over the 2 different focus groups, as aligned with the target population of the diagnostic test packages. Overall, the experiences and choices of the focus groups regarding the website were the same. Therefore, in most cases, the focus group results were discussed together. When the results differed between the 2 groups, this was specified. Different themes around usability, facilitators, barriers, and needs emerged from the data and are elaborated in subsequent sections.

**Table 2:** Characteristics of the participants (N=19).

Participant	Gender	Age (years)	Focus group^a^
1	Woman	27	1
2	Woman	25	1
3	Man	24	1
4	Man	30	1
5	Woman	20	1
6	Woman	25	2
7	Woman	46	2
8	Woman	59	2
9	Man	24	2
10	Man	20	2
11	Woman	25	3
12	Man	25	3
13	Woman	30	3
14	Man	24	3
15	Man	39	4
16	Woman	58	4
17	Woman	59	4
18	Man	30	4
19	Man	62	4

^a^Groups 1 and 3 focused on sexually transmitted infection packages, and groups 2 and 4 focused on prevention packages.

### Facilitators of and Barriers to the Uptake of Innovation

The identified barriers and facilitators were categorized specifically into the following 3 CFIR domains: innovation domain, outer setting domain, and individuals domain. The other 2 domains of the CFIR (ie, inner setting and implementation process) did not align with the facilitators and barriers mentioned by the participants and were therefore not discussed. Table 3 provides insight into the most essential and changeable facilitators and barriers identified. Therefore, it is not an exhaustive list of all potential barriers and facilitators that influenced the service uptake. It is notable that certain factors can be considered as a facilitator and barrier. For example, financial costs are frequently mentioned as a factor affecting the willingness to use digital health services [33]. When there are high user costs, it is a barrier; however, low costs can be considered a facilitator. The identified facilitators and barriers are explained in detail and explained per domain in subsequent sections.

**Table 3:** Facilitators and barriers derived from the focus groups embedded in the Conceptual Framework for Implementation Research (CFIR).

Domain of CFIR			Domain description		Results
**Innovation domain**					
	Innovation source	The group that developed and visibly sponsored the use of the innovation is reputable, credible, and trustable.		The general practitioner group that developed and visibly sponsored the service was reputable, credible, and trustable, which resulted in a reliable service. Information about privacy and presenting good reviews improved reliability and credibility. Commercial appearance influenced the credibility. Furthermore, stock pictures influenced the credibility.	
	Innovation relative advantage	The innovation is better than other available innovations or current practices.		The service was easy to use, which made the service accessible. It was easy to use the service without visiting the general practitioner.	
	Innovation complexity	The innovation is complicated, which may be reflected by its scope and the nature and number of connections and steps.		Too many testing possibilities and too much information made the website less user-friendly. The search bar and filters on the website increased the user-friendliness of the website. Using multiple medical words made the service difficult to comprehend.	
**Outer setting domain**					
	Partnerships and connections	The inner setting is networked with external entities, including referral networks, academic affiliations, and professional organization networks.		The service was linked with academic institutions and other medical professionals, which increased the reliability of the service for users.	
	Societal pressure	Mass media campaigns, advocacy groups, or social movements or protests drive the implementation and delivery of the innovation.		Media campaigns, reviews, and blogs could help stimulate participants to use the service.	
**Individuals domain: subdomain patient characteristics**					
	Capability	The individual has interpersonal competence, knowledge, and skills to fulfill a “Role” (different characteristics of individuals).		If participants had experience with a similar service, they felt more confident in using the service. Otherwise, feelings of anxiety or tension could have influenced their competence, knowledge, and skills.	

### Facilitators and Barriers in the Innovation Domain

#### Innovation Source

Participants mentioned different factors that were related to the innovation source of the innovation domain. These factors mainly influenced the credibility and trustworthiness in a positive (ie, facilitator) or negative (ie, barrier) way. First, the website’s commercial appearance were the most frequently mentioned barriers that influenced its reliability. For example, participants mentioned that the option to buy a gift card for a diagnostic test package did not align with a website designed for health. In addition, regarding the high prices for diagnostic test packages and the website’s general appearance, they said the following:

The website said: buy this. But I want to know why this test?

Participant 4

I found it a very commercial website; this lowers my enthusiasm.

Participant 8

Participants did not notice that health care professionals were involved in the service and partly developed the service, while this could increase the credibility of the website.

Second, the availability of reviews was frequently mentioned as a facilitator for reliability and credibility but as a barrier in some cases. Good reviews could be considered as a facilitator, and bad reviews could be considered as a barrier to experiencing the website as reliable and trustworthy. The following was said about this view:

Yes, [...] I found it important if I go to a new website to sell or buy something to see that others used the site and what they bought.

Participant 13

Third, 37% (7/19) of the participants mentioned the facilitator’s “privacy.” For the participants, it was important to know where the data were stored and for how long. However, this information was difficult to find on the website:

And then it is the question of how long data is stored and how that is important to know.

Participant 8

I want to know, what happens to the data and how long is it stored?

Participant 16

Participant 7 pointed out that a clear and transparent privacy statement could be a unique selling point of the service.

Finally, the most mentioned barrier in the innovation source was the presence of stock pictures on Directlab Online:

[...] those stock pictures on the website; they gave an image of unreliability.

Participant 3

Participants mentioned that pictures of real people or famous people who used the tests could be a facilitator and positively influence the reliability and use of the service. Furthermore, they mentioned that a short video with education and instructions about diagnostic test packages could improve the triage’s clarity and the diagnostic packages’ content.

#### Innovation Relative Advantage

Participants mentioned several factors regarding why they would use this innovative service. These factors were mostly related to the accessibility of the service compared to other services or normal practice. For example, the easiness of ordering a test on the web without going to the general practitioner was a relative advantage of the service, as mentioned by a participant:

Yes, I would rather order online because going to the general practitioner [...] it takes time.

Participant 7

Furthermore, another participant mentioned the benefit of ordering a test on the web without going to the general practitioner:

Hmm yes, I thought of a few things when I first saw the website... of the vitamin tests, STI tests, and COVID tests, I thought yes, you do not want to go to the general practitioner for that. Especially for STI testing, the threshold is high. In this way, you still test and see if you are healthy.

Participant 1

However, the relative advantage was negatively influenced by the high costs of the tests. One participant stated:

The costs will stop people from buying anything.

Participant 17

#### Innovation Complexity

Several facilitators and barriers that influenced the complexity of the service were mentioned by the participants.

First, the number of test packages and parameters available was confusing. It became clear from the focus groups that offering the “right” number of diagnostic tests was important; participants were not enthusiastic about a test package with many separate parameters. Participants mentioned that they were optimistic about the possibility of ordering STI testing, COVID-19 testing, and some prevention tests. However, they mentioned that after the triage, they received advice to select many different test packages. Recommending many diagnostic test packages to the participants was a barrier because they were confused about which test package was important for them. Furthermore, the high amount of information provided about these test packages was experienced as challenging by approximately half (11/19, 58%) of the participants:

When I open the website, a lot of information is present. Too many tests are available. Of course, this website wants to sell tests, but... I do not know. I found the home page too complicated, too unclear.

Participant 13

Second, the language used on the website was a factor that influenced the use of the service. The language on the home page was experienced as straightforward and was therefore a facilitator. However, when completing the triage and choosing the diagnostic package, the information was more challenging to understand. Notably, medical and incomprehensible terms were used:

I think you have a very broad target group of people who would like to use this, and I think it is written for the somewhat well-educated, reasonably well-informed citizen, shall we say [...] Offer more comfort to people by using less difficult vocabulary.

Participant 8

Third, the participants mentioned elements of the website that influenced user-friendliness. Participants were happy with the filters in the search bar to find a particular test, as well as the search function and the website’s colors:

Personally, I found the website easy to use, and what I experienced as very positive were the filters [...].

Participant 14

However, approximately one-third (6/19, 32%) of the participants found the website unclear—among others, due to too much text—and complicated (eg, where to find what they were looking for), and they found the home page too busy.

### Facilitators and Barriers in the Outer Setting Domain

#### Partnerships and Connections

The service was linked to academic institutions, which increased its reliability. Mentioning partners would increase the uptake according to the participants:

Yes, mentioning partners would be nice. And famous names always attract attention.

Participant 13

#### Societal Pressure

Participants mentioned that reviews and blogs could help in increasing the use of the service and its reliability:

You want to read reviews and experiences of others.

Participant 5

### Facilitators and Barriers in the Individuals Domain

The individual’s skills and knowledge regarding services such as Directlab Online influenced their willingness to use the service and their perception of potentially using it. The younger participants (aged 20 to 30 years) mentioned that they had experience with this type of website, which reassured them to use this service. However, some older participants (aged ≥39 years) had less experience with digital services in general and mentioned some anxiety and tension when they needed to order a test. Some (4/19, 21%) preferred to go to the general practitioner for diagnostic tests. However, participants of all age groups mentioned the benefit of ordering STI tests on the web without visiting the general practitioner.

### Future Needs

Different needs were identified regarding services such as Directlab Online. First, the service’s purpose must be more explicit for the participants. For them, it was unclear whether the service could help them self-manage their health:

And this is what I miss on the website; what is in it for me and my health as a patient or consumer?

Participant 19

Second, there was a need to understand the advantages of ordering diagnostic tests on the web (eg, more accessible than going to the general practitioner for tests). Participants wanted this information to be more evident on the website. Third, after receiving their results, the participants explained that they preferred to have more information regarding how they could remain healthy or what they could do to become healthier. It could help, according to the participants, to let them know more specifically that general practitioners make the diagnostic test packages designed for the service. All participants saw the benefit of ordering STI diagnostic test packages on the web and undergoing them at home. The current offer of diagnostic test packages does not meet the wishes of all participants. There was a need for additional tests, such as tests for food allergies, testosterone levels, fertility, or urinary infections. A participant mentioned as follows:

I want a urine tract infection test; those are relatively cheap, I think [...].

Participant 1

## Discussion

### Principal Findings

This qualitative study aimed to evaluate the facilitators of and barriers to web-based direct access to diagnostic test services from the perspective of potential users. In addition, the study tried to identify the need to use such services. The study showed that a tailored amount of information could benefit the service. Participants need to use a service such as Directlab Online to ensure that the website is available for their health. The participants needed to see the benefit of a diagnostic test package. Identified barriers and facilitators were categorized using the CFIR. The study showed that a lack of privacy, information overload, and a commercial appearance were important barriers. Facilitators included providing the right amount of information on the service and involving a health care professional in developing the service. In addition, the study showed that a tailored amount of information could benefit the use of the service. In short, we noticed that several facilitators and barriers were influencing the reliability or accessibility of the service. For example, the commercial appearance and lack of privacy information contributed to a less reliable service for the potential users, and ordering a test on the web without a health care professional influenced the accessibility.

Directlab Online is a service for users to support themselves in self-managing their health. An important quality-enhancing element for Directlab Online was that health care professionals were actively involved in developing this service. Health care professionals significantly influenced the content and information shown on the website. However, the focus groups with potential users identified needs and wishes that did not completely align with the ideas of the general practitioner. To illustrate, health care professionals preferred other types of diagnostic test packages on the web than those that the participants preferred to use. Furthermore, the general practitioners preferred detailed information on the website, whereas this information overload was not always beneficial for the participants. A study by Talboom-Kamp et al [34] regarding a web-based results portal discussed the complex balance between the general practitioner’s necessities and participants’ needs for the right amount of understandable information. Presenting information requires a balance between an overload of medical information and the information users need to understand test packages and results. A potential way to solve overwhelming participants with information is to not present all the information directly in one view to the participant but by offering clickable links or short videos [34].

This study used the CFIR to identify and categorize the facilitators and barriers. In another study, Versluis et al [33] performed an inventory to determine the obstacles that must be overcome and how to optimize eHealth in primary care using this framework. They found similar results to our study; costs and privacy issues were identified as important barriers. In addition, in line with other studies, the following facilitators were identified as having “experience with eHealth” and “easiness of use” [33,35]. In comparison with other studies using the CFIR to classify facilitators and barriers, similar factors were predominantly identified. A notable factor highlighted in the study by Verweij et al [36] involving patients with cancer using a digital self-monitoring system was the necessity to elucidate the service’s added value, alongside concerns regarding privacy issues. However, other factors were also mentioned, such as the connection with health care professionals, which were not identified in our study. The target population (patients with cancer) could be an important explanation for this difference. The comparison with other literature revealed that, irrespective of the type of digital service or the user population, the facilitators and barriers remained quite consistent. This study’s inventory could help determine what obstacles need to be overcome and how we might optimize an application such as Directlab Online.

Depending on the participants, mainly influenced by age, some would use a web-based website to organize their health. In contrast, other participants, mainly older participants, were more at ease with visiting the general practitioner and organizing their health directly via the general practitioner [37]. In this study, the older participants preferred to visit the general practitioner, which could lead to the cautious conclusion that web-based direct access to diagnostic services is not attractive to everyone [37]. In addition, this study showed that using a service such as Directlab Online is not only related to age but also related to the user’s health problem and that the type of test package was important. Participants’ needs were to feel the relevance of ordering a diagnostic test package on the web instead of visiting the general practitioner. The relevance was clear for the STI test packages but unclear for other diagnostic test packages. The study results showed that it remains important to involve all end users in the service to ensure that the service supports the needs of the target population [38]. Directlab Online was developed with general practitioners, and the elements that they found important were integrated into the service. While this study provided insight into the facilitators and barriers of potential users, it appeared that these things were not the same. It is important for a reliable and proper service that both perspectives of all stakeholders should be included in (further) development of such services. Finally, the facilitators and barriers to using a service such as Directlab Online found in this study could be used to optimize this service and other comparable services.

### Strengths and Limitations

There is a lot of direct access to diagnostic testing services available, mainly when it entails STI diagnostic test packages. However, not many of them have a scientific basis or are developed by medical professionals. This is the first study that examined the facilitators of and barriers to a service that provides more diagnostic test packages than only STI tests and which is developed in cocreation with health care professionals. Another strength of the study was that the CFIR was used to analyze the facilitators and barriers mentioned in the focus groups. Embedding the facilitators and barriers in this framework made the comparison with other research easier. In addition, the domains identified by the CFIR can help to find the right implementation strategy [33,39].

This study focused on potential users because we were interested in their first impression of the service. The rationale was that, in the real world, such a service could be visited by many new users [40]. Previous experiences have not biased the impression of potential users. However, this could also be a limitation because participants who did use Directlab Online before could have another opinion regarding the service. This made the results less generalizable. Another limitation is that the mean age of participants was relatively low, making it more difficult to generalize the results to the general Dutch population. However, all participants, independent of age, mentioned the benefit of ordering STI tests on the web. The service showed benefits for participants who were ashamed to visit a general practitioner for a diagnostic package and for participants who wished to order tests in an accessible, nonbinding manner.

### Future Research

Directlab Online is a service developed for a wide range of users. However, this study showed that it is important to include end users to ensure that the service aligns with the population’s needs. Cocreation with end users and medical professionals could be a solution to solve disbalances in wishes and needs between them and to improve an eHealth application [38]. For future research, organizing cocreation sessions and analyzing their results could be beneficial to improve the service. Finally, in future research, information about the influence of the diagnostic test’s result on the user’s lifestyle could be analyzed. Namely, this could result in a preventive role for a service such as Directlab Online to improve the health of a population.

### Conclusions

According to participants, information provision, comprehension, and the overall appearance of the website were the most important elements that influenced the use and uptake of a direct-to-consumer website for diagnostic test packages. Barriers, such as the commercial appearance and lack of privacy information, negatively influenced reliability and accessibility. The study showed that it is important to include relevant stakeholders in creating an eHealth intervention because there was a disbalance between the users’ needs and what the involved general practitioners considered necessary. Future research could take a quantitative approach to further identify the needs regarding test packages and to identify the demographics of users and the influence of test results on the behavior of users. Directlab Online offers opportunities for more web-based self-management of health.

## Appendix

Multimedia Appendix 1 Diagnostic test packages overview of Directlab Online.

Multimedia Appendix 2 Semistructured interview protocol.

## Abbreviations

CFIR: Consolidated Framework for Implementation Research
STI: sexually transmitted infection
