# Supplementary material for: Facilitators of and Barriers to the Use of a Digital Self-Management Service for Diagnostic Testing: Focus Group Study With Potential Users
Source: JMIR Hum Factors. 2024 May 10;11:e45115. doi: 10.2196/45115 (PMC11127139; doi:10.2196/45115)
Supplement: Multimedia Appendix 1 [file humanfactors_v11i1e45115_app1.docx]

| Category | | Parameters |
| --- | --- | --- |
| **Vitamin tests** | |  |
|  | Vitamins check | Gain insight in blood levels of vitamins B6, B11, B12, and D |
|  | Vitamins plus check | Gain insight in blood levels of vitamins B6, B11, B12, D, ferritin and hemoglobin |
|  | Vegetarian | Gain insight in blood levels of vitamins B12, D, hemoglobin, mean corpuscular volume, and ferritin. |
|  | Vegan | Gain insight in blood levels of vitamins B12, D, hemoglobin, mean corpuscular volume and ferritin. |
|  | Vitamin D | Gain insight in vitamin D blood level |
|  | Vitamin B12 | Gain insight in vitamin B12 blood level |
| **Test for common complaints** | |  |
|  | Fatigue | Check for causes of fatigue in blood levels: glucose, thyroid stimulating hormone, C-reactive protein, freeT4, hemoglobin, mean corpuscular volume, ferritin, B11, B12 and glomerular filtration rate |
|  | Hair loss | Check for causes of hair loss in blood levels: hemoglobin, mean corpuscular volume, ferritin, thyroid stimulating hormone) |
|  | Burn out* | Check for causes of burn out in blood levels: glucose (non-fasting), HbA1c, C-reactive protein, thyroid stimulating hormone, freeT4, hemoglobin, mean corpuscular volume, ferritin, Vitamin B11 and B12 |
|  | Why do I not lose weight?* | Check for thyroid stimulating hormone and glucose (non-fasting) |
| Drugs test | |  |
|  | Amphetamine/XTC | Check if there are traces in urine of Amphetamine/XTC |
|  | Benzodiazepines | Check if there are traces in urine of Benzodiazepines |
|  | Cocaine | Check if there are traces in urine of Cocaine |
|  | Cannabis | Check if there are traces in urine of Cannabis |
|  | Opiates | Check if there are traces in urine of Opiates |
|  | Total drugs tests | Check if there are traces in urine of benzodiazepines, amphetamine/XTC, cannabis, cocaine, Gamma Hydroxy Butyrate and opiates. |
| **COVID-19 tests** | |  |
|  | Antibody test | To check if a consumer has antibodies against COVID-19 in their blood |
|  | Post-COVID test | If a consumer has still complaints after a COVID-19 infection he/she can check if something is wrong. Gain insight in blood levels: glucose (non-fasting), total cholesterol, low density lipoproteins, high density lipoproteins, triglycerides, C-reactive protein, thyroid stimulating hormone, freeT4, hemoglobin, mean corpuscular volume, ferritin and vitamins B11, B12 and D |
|  | Vitamins test | Gain insight in blood levels of vitamins B6, B11, B12, and D after COVID-19 infection |

**Appendix 1.** Overview of test packages on Directlab Online

**Table 1.** The other test packages on the website Complete of Directlab Online

*those tests are not available any more on Directlab Online after the service update

| Category | | Parameters |
| --- | --- | --- |
| **Prevention tests** | |  |
|  | Health check-up | Check total cholesterol^a^, low density lipoproteins (LDL)^a^, high density lipoproteins (HDL)^a^, triglycerides^a^, Hba1C^a^, albumin/creatinine ratio^b^. |
|  | Health check-up at home* | Measuring parameters via self-sampling of blood: total cholesterol^c^, LDL^c^, HDL^c^, triglycerides^c^, Hba1C^c^, albumin/creatinine ratio^b^ |
|  | Cholesterol | Check total cholesterol^a^, LDL^a^, HDL^a^, triglycerides^a^ |
|  | Cholesterol at home* | Measuring parameters via self-sampling of blood: Total cholesterol^c^, LDL^c^, HDL^c^, triglycerides^c^ |
|  | Anemia | Check hemoglobin^a^, mean corpuscular volume^a^, ferritin^a^ and C-reactive protein^a^ |
|  | Diabetes | Check glucose^a^ and Hba1C^a^ |
|  | Healthy bones* | Check calcium^a^ and vitamin D^a^ |
|  | Healthy kidneys* | Check creatinine^a^, glomerular filtration rate^a^, albumin/creatinine ratio^b^ |
|  | Thyroid check | Check thyroid function via thyroid stimulating hormone^a^ and freeT4^a^ |
| **Sexual transmitted infections tests** | |  |
|  | Chlamydia | Check for chlamydia^d^ (eg oral, anal, vaginal, urine sample) |
|  | Gonorrhea | Check for gonorrhea^d^ (eg, oral, anal, vaginal, urine sample) |
|  | Human Immunodeficiency Virus (HIV) | Check for HIV^a^ |
|  | Syphilis | Check for syphilis^a^ |
|  | Hepatitis B | Check for Hepatitis B^a^ |

**Table 2.** The test packages focused on in the focus groups

^a^Blood sample needed for diagnostics, ^b^Urine sample needed for diagnostics, ^c^blood sample by self-sampling needed for diagnostics, ^d^Oral, anal, vaginal or urine sample needed for diagnostic tests

*those tests are not available any more on Directlab Online after the service update
