# Supplementary material for: Facilitators of and Barriers to the Use of a Digital Self-Management Service for Diagnostic Testing: Focus Group Study With Potential Users
Source: JMIR Hum Factors. 2024 May 10;11:e45115. doi: 10.2196/45115 (PMC11127139; doi:10.2196/45115)
Supplement: Multimedia Appendix 2 [file humanfactors_v11i1e45115_app2.docx]

**Appendix 2. Semi-structured interview guide**

1. Introduction, explanation, informed consent

a) Welcome. Introduction moderator and note taker

b) Introduction subject

c) Focus group rules

d) Scheduling

e) Consent Form

f) Practical questions?

2. Proposal round

a) Each participant briefly introduces himself.

3. Opening Questions

a) Explanation about digital care in general, Explanation of 'Directlab Online'

b) What were your experiences with digital healthcare before this study started?

Explanation of what we are going to do

*Let the participants go through the website for about 10 minutes.*

4. Overall website

a) How did you find the Directlab website?

b) What is your first reaction to the website?

c) What expectations do you have now? / Is it clear what service is offered on the website?

i) What do you think of the service?

d) How did you experience the website?

i) To what extent did you find the website easy to use?

ii) Do you think you can handle the website quickly?

iii) Were you able to easily find what you were looking for?

iv) To what extent did you find the website attractive?

v) Do you need help using the website?

vi) Does the Directlab website form an unambiguous whole for you?

5. Elements of the website

a) Did you find the general information provided on the website clear?

i) Do you think information is missing?

ii) Do you think other elements are missing on the website (e.g., Chatbot or similar)

b) Have you seen the blogs on the website? If so, will you read or use it?

c) Have you noticed that there are two different types of packages?

i) Yes? Do you understand the difference between the two types of packages? Is a distinction between lifestyle and medical packages of added value for you?

ii) No? Explanation about the two different packages and why it was decided to make this distinction: reliable] How do you view this?

*Show the triage questions yourself, different per focus group*

6. Triage plus test advice

b) How did you experience the questions on the website that led to testing advice?

c) To what extent did you understand these questions?

i) Are there any words you had to look up?

d) To what extent were the questions easy to answer?

e) Did you understand why you had to answer these questions?

7. Facilitators, barriers, improvements: points and potential contributing and counteracting factors of the website and online testing method for the future

a) In principle, this service is intended for everyone. What factors do you think may hinder/encourage the service?

i) Which points do you see as barriers to using Directlab?

ii) The service is currently paid for. Would you pay for it? [Disadvantage, if something is reimbursed, you have to provide more personal information]

iii) Compensation, costs, personal characteristics?

iv) What do you need to assess Directlab (even) more positively?

b) Do you have ideas on how to improve Directlab?

i) If so, what could these improvements look like?

c) Does this way of ordering tests give you a sense of control?

d) To what extent does Directlab feel to you as a reliable service? [probing – why is that]

e) If you were not using Directlab to request diagnostics, would you have gone to the GP?

i) How do you feel about being able to request a diagnostic test without a counselor?

f) How do you experience privacy [complete online questionnaires, order tests, enter personal data, and pay]?

i) How do you think Directlab handles this?

8. Needs: Request utility of online diagnostic test

a) To what extent does this method of ordering online tests meet your needs?

b) Would you use Directlab yourself in the future?

i) What tests would you use Directlab for [tell more about other types of tests]

ii) Would you like to see other types of tests that are not currently available?

iii) Developments are underway about self-drawing blood for a test. How do you feel about this?

c) Would you skip a doctor's appointment using Directlab?

i) How do you feel about being able to request a diagnostic test without a counselor?

9. Closure

a) Of all the things we discussed today, what did you find most important?

b) To what extent would you recommend Directlab to others?

c) Are there any points that we have not discussed?

d) Do you have any additional comments/questions?

e) End of the focus group. Would you like to be kept informed of the results of the research?
